# Molecular docking analysis of antimicrobial peptides with the CXCL1 protein target for colorectal cancer

**DOI:** 10.6026/97320630017369

**Published:** 2021-03-31

**Authors:** Praveen Kumar Kumar, Shanmughavel Piramanayagam

**Affiliations:** 1 Computational Biology Lab, Department of Bioinformatics, Bharathiar University, Coimbatore - 641046, Tamil Nadu, India

**Keywords:** Antimicrobial peptides, colorectal cancer, Peptide docking, Schrodinger

## Abstract

Antimicrobial peptides (AMPs) play a prominent role in drug discovery due to the rapid increase in drug resistant infections. Hence, we report the molecular docking analysis of antimicrobial peptides MREEKKERKRD and MVQGAKRGGRLHRV with the target protein CXCL1 in the context of colorectal cancer for further consideration in drug discovery.

## Background

Colorectal cancer (CRC) is the third most prevalent cancer in the world and causes second mortality rate [1]. Worldwide around 1.4 million people have been identified CRC in the year 2012 [2]. Globally, the incidence of colorectal cancer varies with the highest incidence rates in Australia and New Zealand, Europe and North America and lowest rates recorded in Africa and Asia [3]. Several factors have been shown in individuals at risk to CRC which includes age, presence of polyps, bowel diseases, life style, obesity, poor diet, smoking, alcohol consumption and genetic background have been shown 80% risk of colorectal cancer causes [4]. The most common tumor location in CRC is in the proximal colon, followed by rectum and distal colon [5]. AMPs have a wide spectrum of activities towards different type of organisms such as bacteria, viruses, fungi and mammalian cells, however the molecular mechanism is not yet understood [6]. Conventional treatments like radiation, chemotherapy and surgery are associated with side effects and toxicity, which affects the quality of life. Also, cancer cells are tending to develop resistance against radio and chemotherapy [7]. CRC is responsible for 8.1% of newly diagnosed cases, and 8.3% of all cancer deaths during 2018 [8]. The FDA approved drugs for colorectal cancer are FOLFIRI-CETUXIMAB, FOLFOX, FU- LV, XELIRI, XELOX, Avastin (Bevacizumab), Bevacizumab, Camptosar (Irinotecan Hydrochloride), Capecitabine, Cetuximab, Cyramza (Ramucirumab), Eloxatin (Oxaliplatin), Erbitux (Cetuximab), 5-FU (Fluorouracil Injection), Fluorouracil Injection, Ipilimumab, Irinotecan Hydrochloride, Keytruda (Pembrolizumab), Leucovorin Calcium. Therefore, it is of interest to document the molecular docking analysis of antimicrobial peptides with the CXCL1 protein target for colorectal cancer.

## Methodology

All the computational work has been carried out on Dell Optiplex 380 Intel (R) Core (TM) i-2400 CPU @ 3.10 GHz processor.

## Retrieval of Biological Data:

The 3D structure of the chemokines (C-X-C motif) ligand 1 (CXCL1) was not available in Protein Data Bank. The structure of the CXCL1 was modelled using I-TASSER (Iterative Threading Assembly Refinement) standalone tool [9] with the available amino acid sequence (P09341) from UniProtKB [10]. The best model was taken for further studies. It was validated using the Ramachandran plot generated using the RAMPAGE server [11]. The Antimicrobial peptides were downloaded from NCBI (National Center for Biotechnology Information) and developed the structure by using a molecular graphics tool, PyMOL [12].

## Protein structure preparation:

The protein structure was prepared using protein preparation wizard present in the Maestro 10.2 version of Schrodinger suite [13]. The protein was prepared by adding hydrogen bonds to keep all the atoms in the position and by deleting unwanted chains, removing the water molecules and heteroatom ion compounds. The force field of OPLS_2005 [14] was employed for energy minimization.

## Identification of Active site residues:

Active site residues for the modeled protein structure CXCL1 were identified using the CASTp Server [15]. The residues are 36 SER, 37 VAL, 38 ALA, 39 THR, 40 GLU, 43 CYS, 44 GLN, 45CYS, 46 LEU, 47 GLN, 50 GLN, 51 GLY, 52 ILE, 53 HIS, 54 PRO, 59 SER, 60 VAL, 61 ASN, 62 VAL, 63 LYS, 75 ILE, 76 ALA, 77 THR, 78 LEU, 79 LYS, 82 ARG, 83 LYS, 84 ALA, 88 PRO, 90 SER, 96 ILE, 97 ILE and 98 GLU respectively.

## Receptor grid generation:

Grid generation was carried out using Glide module of Schrodinger suite [16]. Selecting the active sites of the protein generated the grid box. The default grid size of 30 Å x 30 Å x 30 Å was employed for the grid generation.

## Toxicity prediction:

ADMET properties of Antimicrobial peptides were calculated by using the QikProp module [17] in Schrodinger suite. QikProp generates physically relevant descriptors and overall ADME properties and drug-likeness parameter, which were used to assess the drug ability of the compounds as shown in (Table 1).

## Peptide docking:

The peptide structures were prepared by using LigPrep module [18] available in Maestro 11.7 version of Schrodinger suite and the prepared peptides was taken for docking analysis. Two types of docking modes were available in Schrodinger suite, i.e. XP (Extra Precision) mode and SP (Standard Precision) mode. In this study SP (Standard Precision) docking mode was performed for docking analysis with default parameters. The top scored molecular interactions between the protein-peptide complex were described in (Table 2) and the same CXCL1 protein with available small molecule drugs interaction were described in (Table 3).

## Results and Discussion:

**Figure 1:**
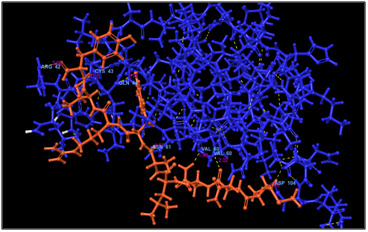
3D docking structure of antimicrobial peptide MNNLAYRTY with CXCl1. Dash lines indicates the hydrogen bond interaction.

**Figure 2:**
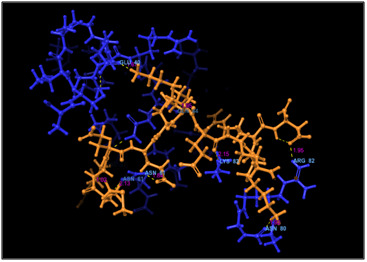
3D docking structure of antimicrobial peptide MREEKKERKRD with CXCl1. Dash lines indicates the hydrogen bond interaction.

**Figure 3:**
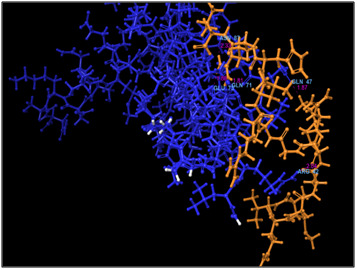
3D docking structure of antimicrobial peptide MVQGAKRGGRLHRV with CXCl1. Dash lines indicates the hydrogen bond interaction.

**Figure 4:**
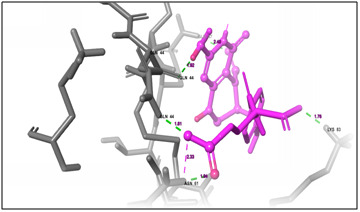
(A) 3D docking structure of CXCl1 (C-X-C Motif Chemokine Ligand 1) protein with the available drug Leucovorin Calcium. The grey colour and sheets indicates the protein structure, pink colour indicates the drug compound and the green dash line indicates the binding affinity between the protein and peptide.

**Figure 5:**
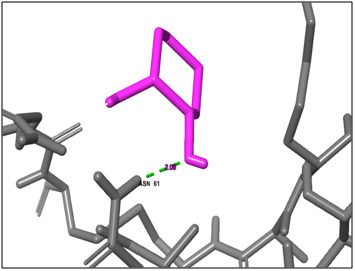
(A) 3D docking structure of CXCl1 (C-X-C Motif Chemokine Ligand 1) protein with the available drug OXALIPLATIN. The grey color and sheets indicates the protein structure, pink color indicates the drug compound and the green dash line indicates the binding affinity between the protein and peptide.

**Figure 6:**
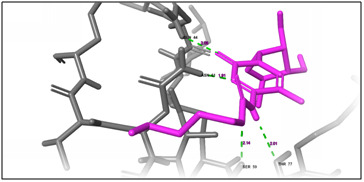
(A) 3D docking structure of CXCl1 (C-X-C Motif Chemokine Ligand 1) protein with the available drug CAPECITABINE. The grey color and sheets indicates the protein structure, pink color indicates the drug compound and the green dash line indicates the binding affinity between the protein and peptide.

**Figure 7:**
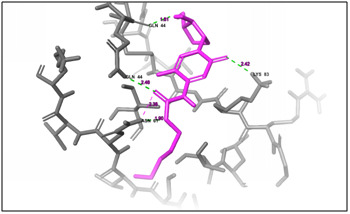
(A) 3D docking structure of CXCl1 (C-X-C Motif Chemokine Ligand 1) protein with the available drug XELOX. The grey color and sheets indicates the protein structure, pink color

Computational docking studies have proven to be useful in the drug discovery and development of small molecule drugs. Similarly there is a rapid growth made in the field of peptide therapeutics. The antimicrobial cleaved peptide sequence with the length of 5-12 amino acids was downloaded and the sequence was converted into structure by using PyMOL. The antimicrobial peptides were screened based on the ADMET toxicity prediction. Those peptides with the CXCL1 target were subjected to molecular docking analysis to find out the best lead drug for colorectal cancer. This study focused on designing a novel peptide drug for colorectal cancer. The binding affinity of the CXCL1 against antimicrobial peptides was determined by molecular docking studies in order to find a lead drug molecule. Computational docking studies identify the top ranked binding affinity of the given antimicrobial peptides with CXCL1 target. The protein-peptides complex got the highest docking score of -57.848, -55.765 shown in (Figure 1, Figure 2, Figure 3) and the FDA approved colorectal drugs has Leucovorin Calcium -5.47, Capecitabine -4.5, XELOX -4.15, Oxaliplatin -4.03, 5-FU (Fluorouracil Injection) -4.11 are shown in (Figure 4, Figure 5,Figure 6,Figure 7) and listed in (Table 3). These results depicts that the target (CXCL1) with the antimicrobial peptide (MREEKKERKRD) complex will acts as a novel lead drug for colorectal cancer.

## Conclusion

We report the molecular docking analysis of antimicrobial peptides MREEKKERKRD and MVQGAKRGGRLHRV with the target protein CXCL1 for colorectal cancer for further consideration in therapy and development.

## Figures and Tables

**Table 1:** ADMET properties of Antimicrobial peptides.

Molecule	Donor HB	Accpt HB	Mol MW	CIQPlogS	QPlogBB	Rule Of Five
ADGTLNEAAIFLM	8.5	31.2	1349.561	-4.145	-15.407	3
AEAMSQVTNSATIM	10	36.3	1437.642	-2.005	-18.637	3
AEGGQA	6.25	16.25	515.522	1.764	-7.212	3
AMLKQLS	7.5	18.2	773.987	-0.521	-7.603	3
ARAVLRGKRM	18.25	26.25	1141.443	-1.924	-17.459	3
ASGRPLA	7.25	17.45	654.765	-0.298	-7.551	3
ASGRPMA	7.25	17.95	672.798	-0.451	-7.36	3
ASGTFSKRIPLA	10.5	28.6	1231.457	-2.897	-15.189	3
ATLNLGHTFGH	8.25	28.15	1151.287	-3.351	-13.014	3
AYANSSNNLE	11.5	29.65	1066.09	0.474	-15.473	3
AYSYNT	7	16.9	701.732	-2.084	-6.386	3
CMIKNLK	9.25	17.75	833.115	-0.586	-7.331	3
CVYSCINLHA	8	23.45	1106.32	-4.716	-9.894	3
DGKVHWWKGI	11.25	23.75	1209.413	-5.505	-11.196	3
DIKNDF	7.75	17.75	734.805	-0.916	-6.607	3
DKYTISL	6.5	17.65	822.954	-2.784	-6.751	3
DRERHIADVGG	15.25	31.25	1208.296	-2.417	-19.02	3
EEHEL	7	18	639.661	-1.381	-7.055	3
EEQVAKFLHII	11.5	30	1310.555	-4.039	-14.499	3
EHEEGGHEI	10	29	1020.021	-1.854	-15.808	3
EKKHCYFYFI	12.25	25.75	1361.62	-7.972	-11.451	3
EMNLKEIK	11.5	24	988.207	-0.307	-11.772	3
ETILNFGENL	9.75	27.95	1133.263	-2.318	-12.174	3
EYILEN	8	18.75	763.843	-2.119	-7.839	3
FASNNIIK	9.25	21.45	890.047	-0.414	-9.386	3
FNCFPY	5.75	16.45	773.902	-3.911	-3.452	3
FSHDCNLVNFL	9.75	28.95	1292.471	-5.081	-12.939	3
GAAREGAGGFEV	10.75	28.25	1104.185	-1.524	-17.862	3

**Table 2:** Molecular Interaction results of antimicrobial peptides with CXCL1

Name of the compound	Docking Score	Glide Score	Potential Energy
MREEKKERKRD	-57.848	-57.848	-4331.582
MVQGAKRGGRLHRV	-55.765	-55.765	-2888.939
MNNLAYRTY	-10.32	-10.32	-1573.344
MAGGYASDSDNESEDDD	-9.75	-9.75	-4889.139
LITKERFESMSN	-9.718	-9.718	-2654.931
MEGVAERMNRTIVEKM	-9.521	-9.521	-3861.543
YQLIIQEDMTL	-9.505	-9.505	-2333.009
MIALFDTSTDLNCI	-9.482	-9.482	-2374.991
MVGKLTYT	-9.425	-9.425	-1089.211
MMMAMLCWDNQKDVK	-9.382	-9.382	-3353.819
MSEIVREARA	-9.375	-9.375	-2027.248
MGVFCWITNYFREDEG	-9.348	-9.348	-3330.243
MNINDEKTAHLAV	-9.265	-9.265	-2797.957
EYILEN	-9.21	-9.21	-1436.889
MAGGYASDSDNESEDDD	-9.195	-9.195	-4889.139
CFGIAGFELALWHD	-9.121	-9.121	-2402.832
MTNLAYKTYNIES	-9.114	-9.114	-2410.694
CFGIAGFELALWHD	-9.089	-9.089	-2402.832
SKDTNFLNGFGVQV	-9.083	-9.083	-2646.398
MAERSEAKSA	-8.984	-8.984	-2107.11
METKHDVKHIK	-8.968	-8.968	-2445.825
MNEINQQ	-8.961	-8.961	-1730.656
CFGIAGFELALWHD	-8.933	-8.933	-2402.832

**Table 3:** Molecular Interaction results of available colorectal drugs with CXCL1

Name of the Drugs	GScore	Dock Score	Hbond
Leucovorin Calcium	-5.47	-5.47	-2.46
Capecitabine	-4.5	-4.5	-3
Xelox	-4.15	-4.15	-2.31
Oxaliplatin	-4.03	-4.03	-0.35
